# Curcumin-Mediated Degradation of S-Phase Kinase Protein 2 Induces Cytotoxic Effects in Human Papillomavirus-Positive and Negative Squamous Carcinoma Cells

**DOI:** 10.3389/fonc.2018.00399

**Published:** 2018-10-02

**Authors:** Abdul Q. Khan, Kodappully S. Siveen, Kirti S. Prabhu, Shilpa Kuttikrishnan, Sabah Akhtar, Abdullah Shaar, Afsheen Raza, Fatima Mraiche, Said Dermime, Shahab Uddin

**Affiliations:** ^1^Translational Research Institute, Academic Health System, Hamad Medical Corporation, Doha, Qatar; ^2^National Center for Cancer Care and Research, Hamad Medical Corporation, Doha, Qatar; ^3^School of Pharmacy, Qatar University, Doha, Qatar

**Keywords:** cancer, head and neck squamous cell carcinoma, HPV, Skp2, apoptosis

## Abstract

S-phase kinase-associated protein2 (Skp2), a proto-oncoprotein, plays an important role in development and progression of human malignancies. Skp2 is frequently overexpressed in many human malignancies. It targets cell cycle progression through ubiquitin mediated degradation of G1-checkpoint CDK inhibitors—p21 (CDKN1A) and p27 (CDKN1B). We investigated the role of Skp2 and its ubiquitin-proteasome pathway in head and neck squamous cell carcinoma (HNSCC) using a panel of cell lines with and without human papillomavirus (HPV^+^, HPV^−^). Treatment of HNSCC cell lines with curcumin, a natural compound isolated from rhizomes of the plant *Curcuma longa*, or transfection of small interfering RNA of Skp2, causes down-regulation of Skp2 with concomitant accumulation of p21 and p27 in HPV^+^, HPV^−^ cells. Furthermore curcumin inhibits cell viability and induces apoptosis in a dose-dependent manner. Treatment of HPV^+^ and HPV^−^ cells with curcumin induced apoptosis via mitochondrial pathway and activation of caspases. In addition, treatment of HPV^+^ and HPV^−^ cell lines with curcumin down-regulated the expression of XIAP, cIAP1, and cIAP2. Interestingly, co-treatment of HNSCC cells with curcumin and cisplatin potentiated inhibition of cell viability and apoptotic effects. Altogether, these data suggest an important function for curcumin, acting as a suppressor of oncoprotein Skp2 in squamous cell carcinoma cells in both HPV^+^ and HPV^−^ cells; raise the possibility that this agent may have a future therapeutic role in squamous cell carcinoma.

## Introduction

Cancer is a complex, life-threatening disease and one of the leading causes of morbidity and mortality around the world (1). Head and neck cancer (malignancy of oral cavity, oropharynx, hypopharynx, and larynx) is the sixth most commonly diagnosed and ninth leading cause of cancer-related death in humans (2, 3). Use of alcohol and tobacco-related products are the major etiological factors for the malignancies associated with head and neck, and squamous cell carcinoma (HNSCC) is the most common (3).

Human papillomavirus (HPV) has been recognized as an independent risk factor for such tumors, especially if the tumor is located in the oropharynx, where about 50% of tumors harbor the virus (4–7). The HPV status has showed a significant role on prognosis: recent studies revealed that HPV positive (HPV^+^) patients have a better prognosis as compared to HPV negative (HPV^−^) patients (8). It is now well established that HPV^+^ tumors are distinct tumor entity in regards to carcinogenesis and mutational status as compared to HPV^−^ HNSCC (9). Due to the better prognosis, HPV^+^ tumor cells possess intrinsic properties including an increased sensitivity to therapeutic agents, suppressed proliferation rate, and a better immune response due to the presence of the virus.

Currently, available treatments such as tumor surgery, chemotherapy, radiotherapy, or combinational therapy are associated with number of complications which entails the need of further research for better therapeutic outcomes (10). Identification of the critical drug targets is imperative for a positive outcome of cancer treatment. In the current study, we have elucidated that S-phase kinase-associated protein 2 (Skp2) could be a potential target for the treatment of head and neck cancer by using curcumin, a natural compound isolated from rhizomes of the plant *Curcuma longa*, and is the most commonly used food additive with strong anti-oxidant, anti-inflammatory, anti-microbial, hypoglycemic, and wound-healing activities (11). Various findings demonstrated the clinical importance of curcumin at preclinical and clinical levels against a number of human pathological conditions including cancer most likely attributed to its pleiotropic therapeutic targets/ signaling machinery (12, 13). Considering the clinical importance of curcumin, more research work needs to be done to understand the underlying mechanism of its anticancer potential.

Skp2, a proto-oncogenic F-Box protein of SCF E3 ubiquitin ligase complex, play critical role in carcinogenesis as it targets cell cycle progression through degradation of specific targets such as G1-checkpoint CDK inhibitors-p21 (CDKN1A) and p27 (CDKN1B), p57, and foxo1 (forkhead box O1) (14, 15). Potential of the role of Skp2 in the sequential and stepwise development of cancer is well elucidated. Oncogenic role of Skp2 has been well documented that it downregulates expression of cell cycle inhibitory proteins such as p21, p27, and foxo1 through proteasomal degradation (14). Poor prognosis of human malignancies has been associated with Skp2 overexpression (16, 17). Furthermore, it has been reported that AKT/PKB, a vital signaling protein, mediated tumorigenesis involves interaction and phosphorylation of Skp2 (18). Oncogenic role of Skp2 is well established in the malignancies of head and neck ranging from squamous cell carcinoma to melanoma (18–22). Therefore, in the current study, we have elucidated the clinical relevance of curcumin for cancer treatment on a panel of human head and neck cancer cell lines by targeting deregulated overexpression of Skp2 and associated signaling components.

In this study, we investigated the effects of curcumin on HPV^+^ and HPV^−^ cell lines focusing on cytotoxicity effects. Our data showed curcumin treatment of HNSCC cell with and without HPV status suppressed the viability via induction of apoptosis. Curcumin treatment of HPV^+^ and HPV^−^ cell lines downregulated the expression of Skp2 with concomitant upregulation of p27 and p21. Furthermore, curcumin induced apoptosis involves mitochondrial and caspase-cascade signaling pathway in both types of HNSCC cells. In addition, curcumin potentiated the effects of cisplatin-induced anticancer effects in these cells. These data suggest that curcumin-induces anticancer effects in HNSCC independent of HPV^+^ and HPV^−^ status.

## Materials and methods

### Reagents and antibodies

Curcumin, cycloheximide, and cisplatin were purchased from Sigma Aldrich (St. Louis, Missouri, United States). Antibodies against caspase-9, caspase-8, caspase3, cleaved caspase-3, PARP, XIAP, cIAP1, cIAP, Bcl2, Bclxl, Skp2, p27, p27, tubulin, ubiquitin, etc., were purchased from Cell Signaling Technologies (Beverly, MA, USA). GAPDH antibody was purchased from Santa Cruz Biotechnology, Inc. (Santa Cruz, CA, USA). Annexin V-FITC, Propidium iodide staining solution, Hoechst 33342 Solution, BD Cytofix/Cytoperm plus fixation and permeabilization solution kit, BD MitoScreen (JC-1) Kit, were purchased from BD Biosciences (NJ, USA).

### Cell culture

A panel of human head and neck cancer cell lines (SCC25, FaDu, and SCC090) were cultured using RPMI 1640 medium supplemented with 10% (v/v) fetal bovine serum (FBS), 100 U/ml penicillin and 100 U/ml streptomycin at 37°C in a humidified atmosphere containing 5% CO_2_.

### Measurement of real time cell proliferation of HNSCC cells treated with curcumin using RTCA; xCELLigence cell analyzer

SCC25, FaDu, and SCC090 cells were grown in monolayer on top of the electrodes and were treated with different doses (10, 20, 40 μM)of curcumin. The real time cell analyzer and E-plate 16 (RTCA; xCELLigence, Roche, San Diego, CA, USA) was used to determine the cell viability of curcumin treated and untreated cultured cells using electrical impedance (23).

### Cell counting Kit-8 (CCK8) assay

The anti-proliferative effects of curcumin, HNSCC cell lines (SCC25, FaDu, and SCC090), was performed by using cell counting kit-8 reagent as described previously (24). Briefly, 10^4^ cells were incubated in a 96-well plate and treated with five different doses (5, 10, 20, 40, and 80 μM) of curcumin for 24 h. Furthermore, we have treated FaDu cells with curcumin and cisplatin alone and in combination for 24 h at 37°C. After that, cell counting Kit-8 solution was added as per the manufacturer's instruction followed by incubation at 37°C. Finally, the optical density (OD) was recorded at 450 nm. Percent cell viability was calculated as OD of the experiment samples/OD of the control sample × 100.

### Annexin V/propidium iodide staining

HNSCC cell lines were treated with three different concentrations of curcumin followed by incubation for 24 h. Cells were harvested, washed with PBS, and then stained with fluorescein-conjugated Annexin V and Propidium Iodide. Finally, apoptosis was measured as described previously (25, 26) by flow cytometry (BD LSRFortessa analyzer, BD Biosciences).

### Cell lysis and immunoblotting

Following the treatment with varying doses of drugs and inhibitors, cells were harvested and lysed as described previously (27). An equal amount of proteins were separated on SDS-PAGE and transferred to polyvinylidene difluoride (PVDF) membrane. Specific antibodies were used against proteins of interest and were immunoblotted and further visualized under a ChemiDoc System (Amersham, Bio-Rad, USA).

### Measurement of mitochondrial membrane potential

Appropriate numbers of cells were treated with gradient doses of curcumin for 24 h. After the treatment, cells were harvested, washed, and finally stained with JC1 stain kit as per the manufacturer's instruction and then analyzed using flow cytometry (BD LSR Fortessa analyzer, BD Biosciences, USA).

### Assay for cytochrome C release

SCC25, FaDu, and SCC090 cells were plated and then treated with different concentration of curcumin for 24 h, then cells were harvested and resuspended in hypotonic buffer. The mitochondrial and cytosolic fraction was isolated as described earlier (28). Protein concentration in cytosolic fraction of each sample was measured and analyzed by immunoblotting using an anti-cytochrome c and GAPDH antibody.

### Gene silencing using siRNA

Skp2 siRNA (catalog no. S102659692, Batch no. 289614, Batch as. 289615) and scrambled control siRNA (catalog no.1027281, Lot no.190563210) were obtained from Qiagen. FaDu and SCC090 cells were transfected using Lipofectamine 2000 reagent (Invitrogen) according to the manufacturer's instructions. The lipid and siRNA complex was removed after 6 h, cells were supplemented with complete medium and incubated for 48 h. Cells were lysed and immunoblotted with various antibodies.

### Statistical analysis

The data from individual groups were presented as the means ± standard deviation (SD). Comparison between groups was made using one way analysis of variance (ANOVA) followed by *Tukey-Kramer* multiple comparisons test. The software GraphPad Prism (version 5.0 for Windows, GraphPad Software Inc., San Diego, CA, http://www.graphpad.com) was used. Values of ^*^p < 0.05, ^**^p < 0.01, and ^***^p < 0.001 were considered statistically significant.

## Results

### Curcumin inhibits cell viability of HPV^+^ and HPV^−^ HNSCC cell lines through apoptosis

**Figure 1:**
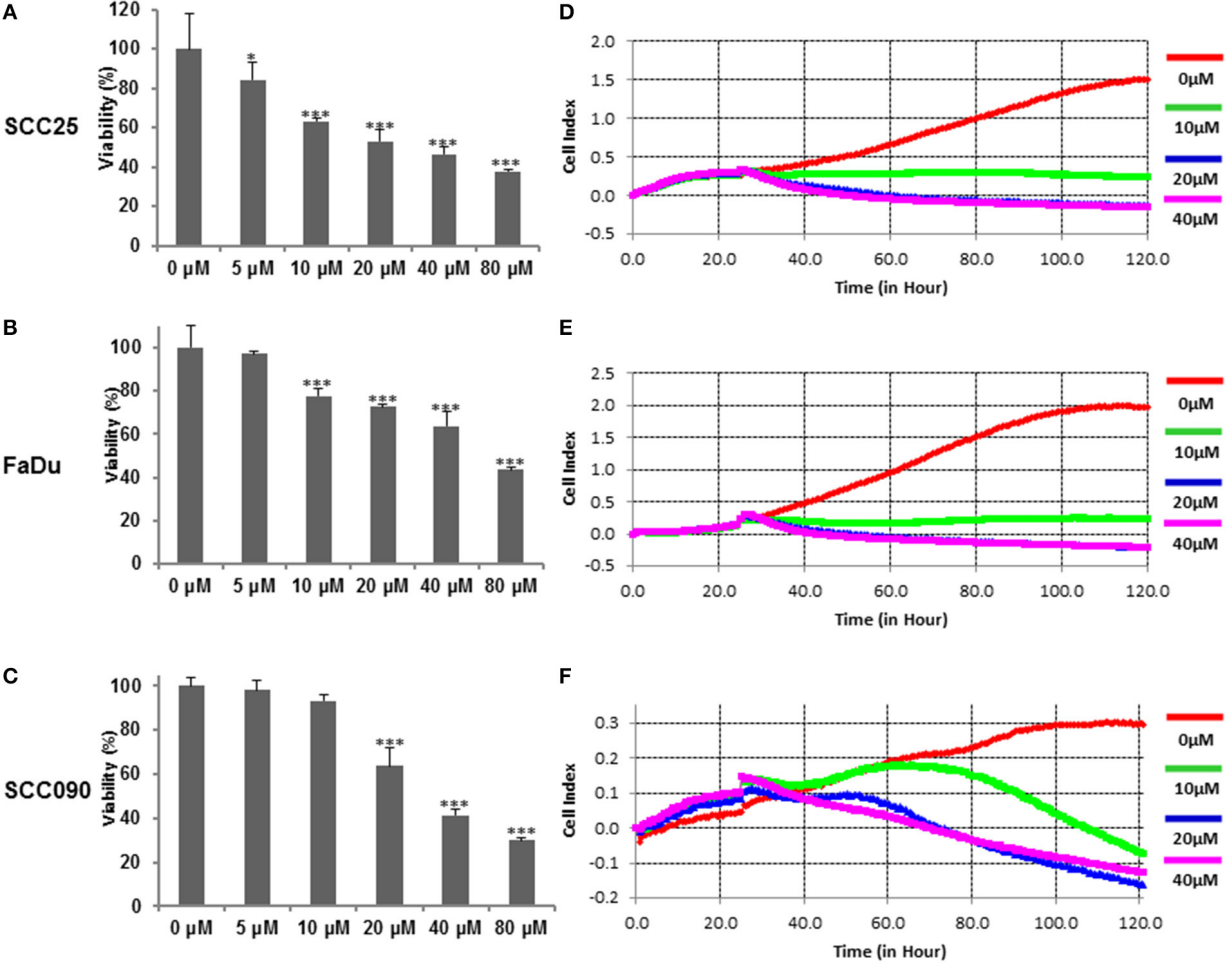
Curcumin suppresses dose-dependent cell proliferation in HNSCC cells. Curcumin inhibits the cell viability of HNSCC cells. **(A)** SCC25 **(B)**, FaDu, and **(C)** SCC090 cells were incubated with 5, 10, 20, 40, and 80 μM curcumin for 24 h. Cell proliferation assay was performed using CCK8 as described in Materials and Methods. The graph displays the mean ± S.D. (standard deviation) of three independent experiments with replicates of six wells for all the doses. ^*^p < 0.05, ^***^p < 0.001. Real time cell proliferation (cell index) analysis of HNSCC cells. **(D)** SCC25 **(E)** FaDu, and **(F)** SCC090, cell were grown in monolayer on top of the electrodes and treated with indicated concentration of curcumin. The real time cell analyzer was used to determine cell index as described in method section.

We initially sought to determine the effects of curcumin on cell viability on HPV^−^ (SCC25 and FaDu), and HPV^+^ (SCC090) HNSCC cell lines. The respective HNSCC cells were treated with increasing doses of curcumin for 24 h and cell viability of treated and untreated cell lines was assayed using CCK8. Results and data analysis revealed that curcumin inhibited cell viability in a dose-dependent manner in all cell lines irrespective of HPV status (Figures 1A–C). To determine the real time cell proliferation in response to curcumin treatment of HPV^−^ and HPV^+^ HNSCC cell lines, xCELLigence Real-Time Cell Analysis (RTCA) was performed on HNSCC cell lines. RTCA results showed that curcumin induces a dose and time dependent inhibition of cell proliferation in all HNSCC cell lines (Figures 1D–F).

**Figure 2:**
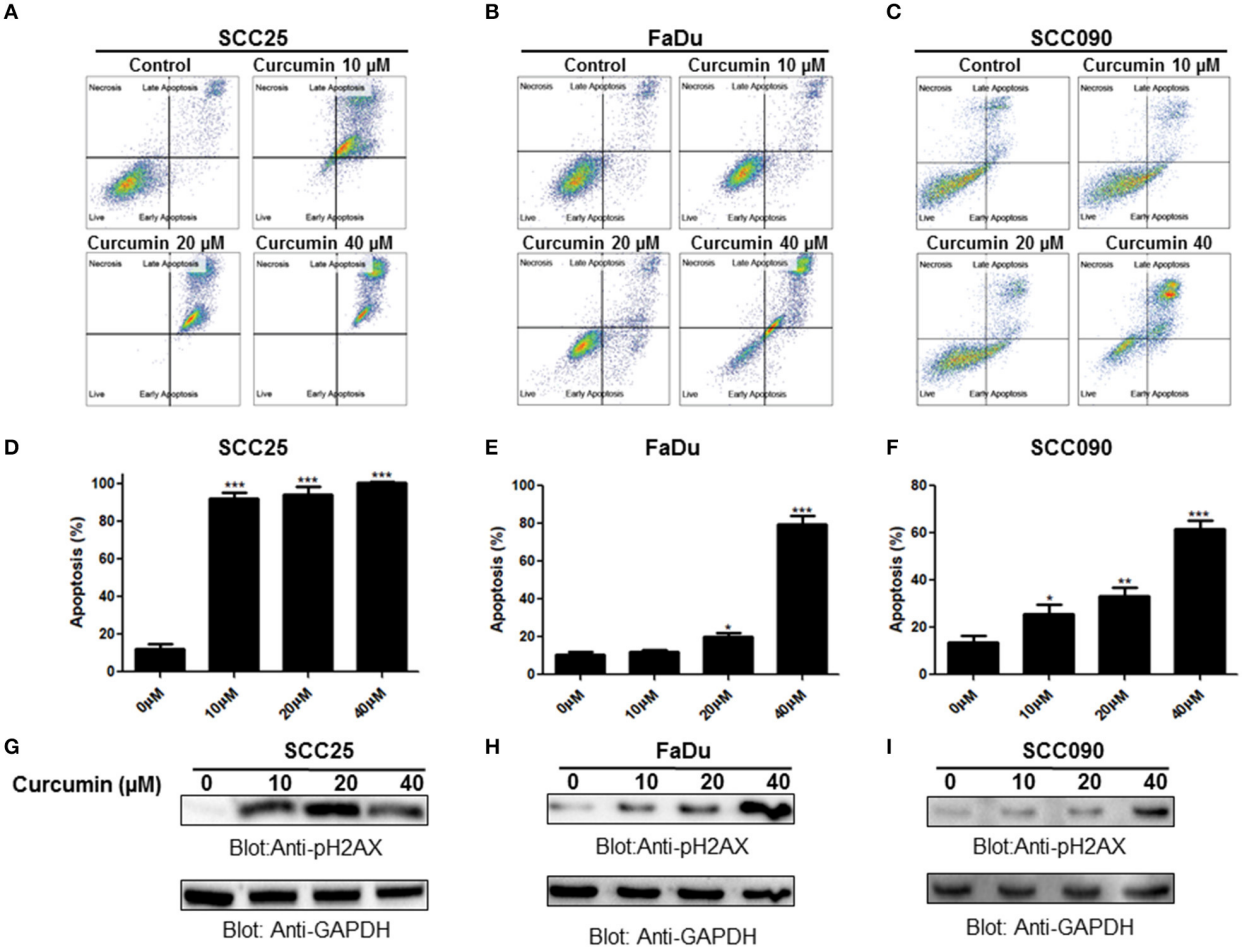
Curcumin-induced apoptosis in HNSCC cells. Curcumin mediated annexin/PI staining in HNSCC cells. **(A)** SCC25, **(B)** FaDu, and **(C)** SCC090 cells were treated with 10, 20, 40 μM curcumin for 24 h and cells were subsequently stained with flourescein-conjugated annexin-V and propidium iodide (PI) and analyzed by flow cytometry. Curcumin mediated apoptosis in HNSCC cell line. **(D)** SCC25, **(E)** FaDu, and **(F)** SCC090 cell line were treated with curcumin and apoptosis was measured by flow cytometry after staining with annexin-V and PI. Percentage of apoptosis relative to untreated cells was calculated as described previously (26). The graph displays the mean ± S.D. of three independent of experiments. ^*^p < 0.05, ^**^p < 0.01, ^***^p < 0.001. Curcumin mediated phosphorylation of H2AX in HNSCC cell lines. **(G)**SCC25, **(H)** FaDu, and **(I)** SCC090 cells were treated with 10, 20, 40 μM curcumin for 24 h and cells were lysed. After lysis proteins were separated by SDS–PAGE, transferred to PVDF membrane and immunoblotted with p-H2AX and GAPDH antibodies as indicated.

In the subsequent experiment, we determined whether curcumin-mediated inhibition of cell viability is due to apoptotic cell death. We performed annexin V/PI dual staining on curcumin treated SCC25, FaDu, and SCC090 cell lines. As shown in Figures 2A–C curcumin treatment resulted in the increase in a dose-dependent manner of annexin-V/PI staining. Curcumin significantly induced apoptosis at 10 μM and above concentration in SCC25 and SCC090. However in FaDu curcumin was found to cause significant apoptosis at 20 μM and above dose (Figures 2D–F). In addition, curcumin treatment caused dose-dependent increase in phosphorylation of H2AX (Figures 2G–I) which indicates double-stranded DNA breaks (Supplementary Figures 1A–C). These results suggest that after curcumin treatment, inhibition of cell viability in HNSCC cells occur due induction of apoptosis.

### Curcumin inhibits proteasomal activity via degradation of Skp2 in HNSCC cells

**Figure 3:**
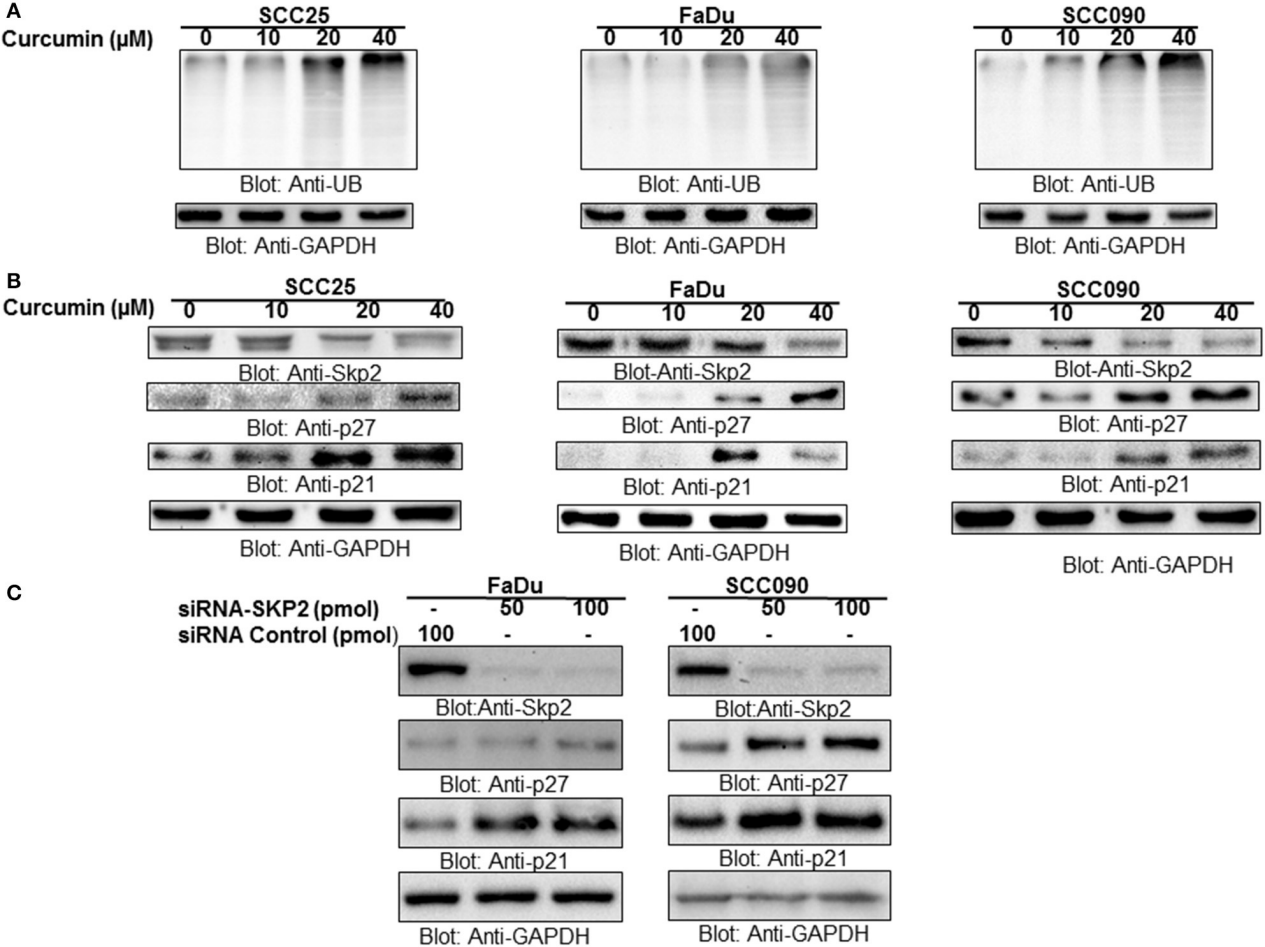
Curcumin mediated accumulation of ubiquitinated proteins via suppression of F-box protein Skp2 in HNSCC cell lines **(A)** Curcumin-mediated ubiquitination of various proteins. SCC25, FaDu, and SCC090 cells were treated with indicated doses of curcumin for 24 h. Equal amounts of protein lysates were separated by SDS-PAGE, transferred to PVDF membrane, and immunoblotted with antibodies of anti-ubiquitin and GAPDH as indicated **(B)** Curcumin treatment down-regulated the expression of Skp2 and enhanced the level of p27 and P21. SCC25, FaDu, and SCC090 cells were treated with various doses of curcumin for 24 h. After cell lysis, equal amounts of proteins were separated by SDS-PAGE, transferred to PVDF membrane, and immuno-blotted with antibodies against Skp2, p27, p21, and GAPDH as indicated **(C)** Skp2 siRNA transfection downregulates Skp2 and accumulated p27 and p21. FaDu and SCC090 cells were transfected with Scrambled siRNA (100 pmol) and Skp2 siRNA (50 and 100 pmol) using Lipofectamine 2000 as described in Materials and Methods. After 48 h of transfection, cells were lysed and equal amounts of proteins were separated by SDS-PAGE, transferred to PVDF membrane, and immunoblotted with antibodies against Skp2, p27, p21, and GAPDH as indicated.

Cyclin-dependent kinases are the major regulatory proteins critically associated with cell proliferation and growth, and their action is precisely controlled by inhibitory proteins such as p27 and p21 via ubiquitination and proteasomal degradation. Skp2, an integral substrate recognizing the protein in SCF (Skp1-Cullin1-F-box) E3 ubiquitin-ligase complex, play critical role in oncogenesis via ubiquitin-mediated proteasomal degradation of a number of signaling proteins including p27 and p21. Keeping above facts in consideration, in the present study, we determined the role of Skp2 degradation/downregulation and proteasomal ubiquitination in curcumin-mediated apoptosis in HNSCC cell lines treated with curcumin. It was observed that HNSCC treatment with curcumin led to the accumulation of polyubiquitinated proteins most likely via inhibition of proteasome in HPV^+^ and HPV^−^ HNSCC cells (Figure 3A). Interestingly, there was a dose-dependent downregulation of Skp2 with concomitant increased in expression of cyclin-dependent kinase inhibitors p27 and p21 seen after curcumin treatment in all cell lines (Figure 3B, Supplementary Figures 1D–F). In addition, immunostaining of SKP2 on curcumin treated HNSCC cell lines showed a decreased staining (Supplementary Figure 2). These findings suggest that there is an inverse biological functional link between Skp2 and cell cycle proliferation. Furthermore, the antagonistic action of curcumin for Skp2 and cell cycle inhibitor proteins suggests that curcumin-mediated apoptosis in HNSCC cells most likely occurs through Skp2 mediated upregulation of p27 and p21.

Curcumin-mediated accumulation of p27 prompts us to investigate further the effect of curcumin on the stability of p27 using cycloheximide chase assay at different time intervals. As shown in Supplementary Figure 2B, compared to untreated cells, curcumin treatment of FaDu cells stabilized the expression of p27 indicating that curcumin-mediated upregulation of p27 is due to the stabilizing effect of curcumin on p27.

To further confirm the antagonistic effect observed for Skp2, p27, and p21, we performed gene silencing experiments using HPV^−^ FaDu, and HPV^+^ SCC090 cell lines. Cells were transfected with Skp2 specific siRNA and expression of Skp2, p27, and p21 were determined by immunoblotting with antibodies against Skp2, p27, and p21. As shown in Figure 3C, knockdown of Skp2 resulted in the increased expression of p27 and p21 in both HPV^+^and HPV^−^ HNSCC cells. Keeping these results in perspective, it is suggested that the curcumin-induced apoptosis in HNSCC cells is mediated by the downregulation of Skp2 and concomitant accumulation of p27 and p21.

### Curcumin treatment suppresses Bcl-2 expression and enhances Bax expression in HNSCC cells

**Figure 4:**
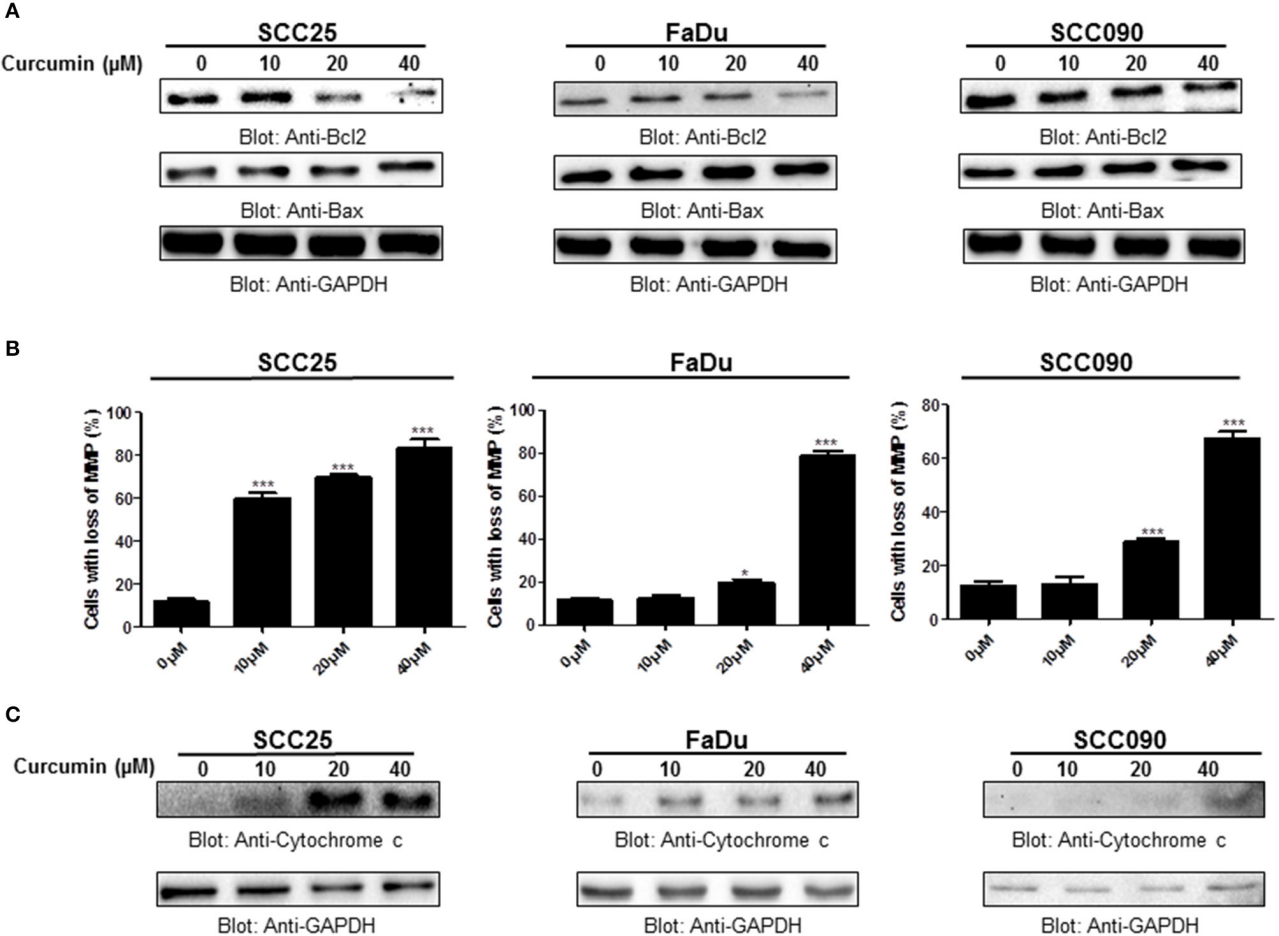
Curcumin-induced activation mitochondrial apoptotic pathway. **(A)** Curcumin-mediated upregulation of Bax expression and downregulation of Bcl2 in HNSCC cells. SCC25, FaDu, and SCC090 cells were treated with indicated doses of curcumin for 24 h. Equal amounts of protein lysates were separated by SDS–PAGE, transferred to PVDF membrane and immunoblotted with Bax, Bcl2, and GAPDH antibodies as indicated. **(B)** Curcumin treatment causes the loss of mitochondrial membrane potential in HNSCC. SCC25, FaDu, and SCC090 cells were treated with indicated doses of curcumin for 24 h. After JC1 staining cells were analyzed by flow cytometry as described in Materials and Methods. The graph displays the mean ± S.D. of three independent of experiments. ^*^p < 0.05, ^***^p < 0.001. **(C)** The curcumin-induced release of cytochrome c. SCC25, FaDu, and SCC090 cells were treated with and without curcumin for 24 h. Cytoplasmic fraction was isolated as described in Materials and Methods. Cell extracts were separated on SDS-PAGE, transferred to PVDF membrane, and immunoblotted with an antibody against cytochrome c and GAPDH.

Bcl-2 family members play a significant and pivotal role in regulating apoptosis by maintaining a balance between anti-apoptotic molecules such as Bcl-2 and pro-apoptotic molecule Bax. Imbalance or disturbance in these proteins levels leads to stimulation or prevention of cell death. We aimed to determine whether treatment of HNSCC cells with curcumin enhances the expression levels of Bax and suppress the expression of Bcl-2. As shown in Figure 4A, it is observed that treatment of HNSCC cells with curcumin caused a decrease in expression levels of anti-apoptotic Bcl-2 protein with a subsequent increase in expression level of pro-apoptotic protein Bax indicating that curcumin-mediated expression of Bax and downregulation of Bcl2 play a role in curcumin induced apoptosis. Low Bax and high Bcl-2 expression has been shown to cause resistance whereas as high level of Bax and low Bcl-2 expression is found to result in sensitivity to drug-induced apoptosis. Our data showed that curcumin treatment of HNSCC cells caused an increased level of Bax expression and decreased expression of Bcl2 suggesting that curcumin-mediated expression of Bax and downregulation of Bcl2 play a role in curcumin induced apoptosis.

### Curcumin-mediated apoptosis involves activation of the intrinsic mitochondrial apoptotic pathway and caspases activation

**Figure 5:**
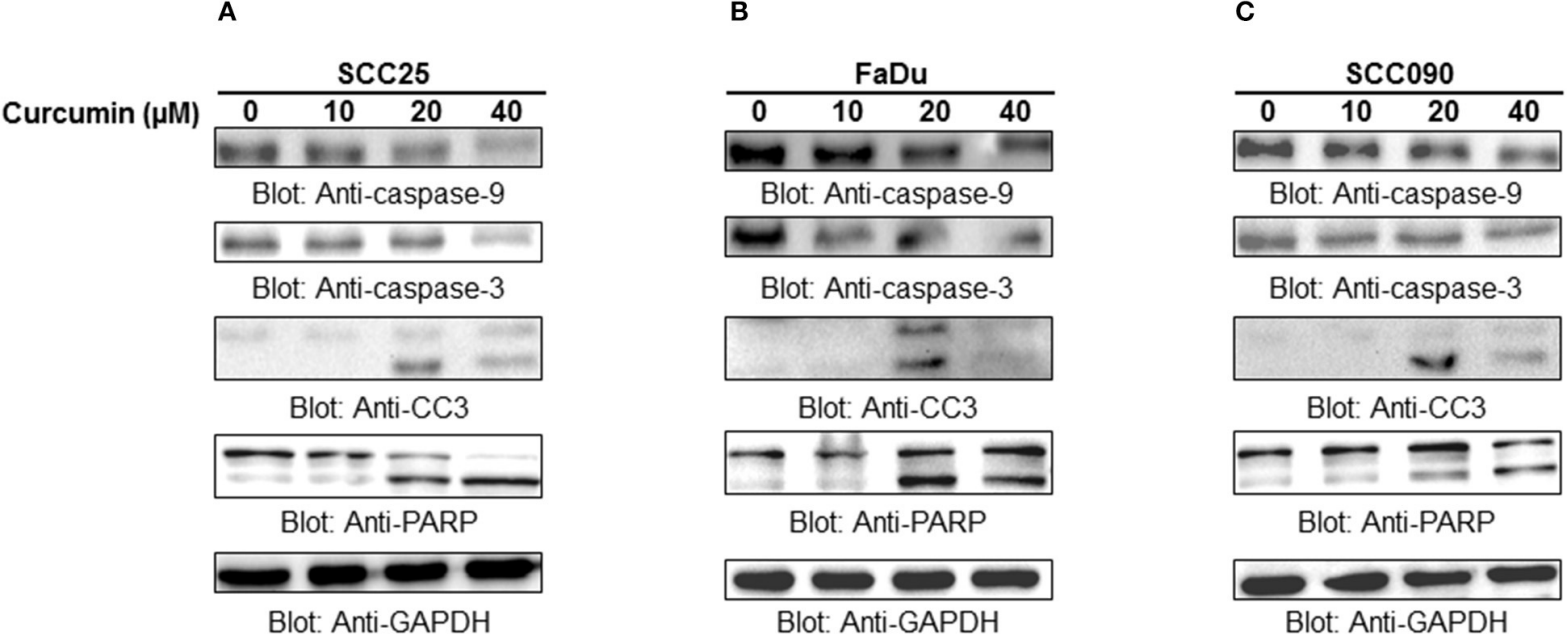
Curcumin-mediated activation of caspases in HNSCC cells: **(A)** SCC25, **(B)** FaDu, and **(C)** SCC090 cell lines were treated with indicated doses of curcumin for 24 h. Equal amounts of protein lysates were separated by SDS–PAGE, transfered to PVDF membrane, and immunoblotted with antibodies of caspases, and GAPDH as indicated.

Apoptosis is a complex physiological phenomenon, and a number of factors are known to play a vital role in natural cell death. Here in the current study, we studied the mechanism underlying curcumin-induced apoptosis with a number of convergent apoptotic markers. We sought to determine, whether curcumin-induced apoptosis involves mitochondrial-mediated activation of caspases. For this, curcumin treated cells were labeled with JCI dye and subjected to flow cytometry for MMP analysis. Our results showed that treatment of HNSCC cells with curcumin resulted in increased JC1 staining indicating loss of MMP in a dose-dependent manner. As shown in Figure 4B, control cells showed JCI-aggregate complex with no or slight reduction in MMP while in case of curcumin-treated cells apoptosis was observed as indicated by a significant reduction in MMP. Furthermore, we observed that curcumin treatment to HNSCC cell lines induces the release of mitochondrial cytochrome c into the cytosol (Figure 4C). We sought to determine whether this released cytochrome c leads to activation caspase-cascade. As shown in Figures 5A–C, we observed that treatment of HNSCC cell lines with curcumin resulted in activation of caspase-9 with subsequent activation of caspase-3 and cleavage of PARP in a dose dependent manner.

Inhibitors of apoptosis proteins (IAPs) have been shown to play a critical role on the activity of caspases. We, therefore, sought to determine whether curcumin-mediated apoptosis occurs via involving IAP members. SCC25, FaDu, and SCC090 cells were treated with curcumin and expression of XIAP, cIAP1, and cIAP2 were determined by immunoblotting using antibodies against these IAPs. As shown in Supplementary Figure 3, curcumin treatment resulted in down-regulation of XIAP, cIAP-1, and cIAP2 in a dose-dependent manner. Indicating that curcumin-mediated apoptosis involves these XIAP, cIAP-1, and cIAP2 proteins in HNSCC cells. Altogether, these results suggest that curcumin-mediated cytotoxic effects in HNSCC cells is due to activation of mitochondrial and caspase-cascade.

### Curcumin synergistically potentiates the chemotherapeutic action of cisplatin

**Figure 6:**
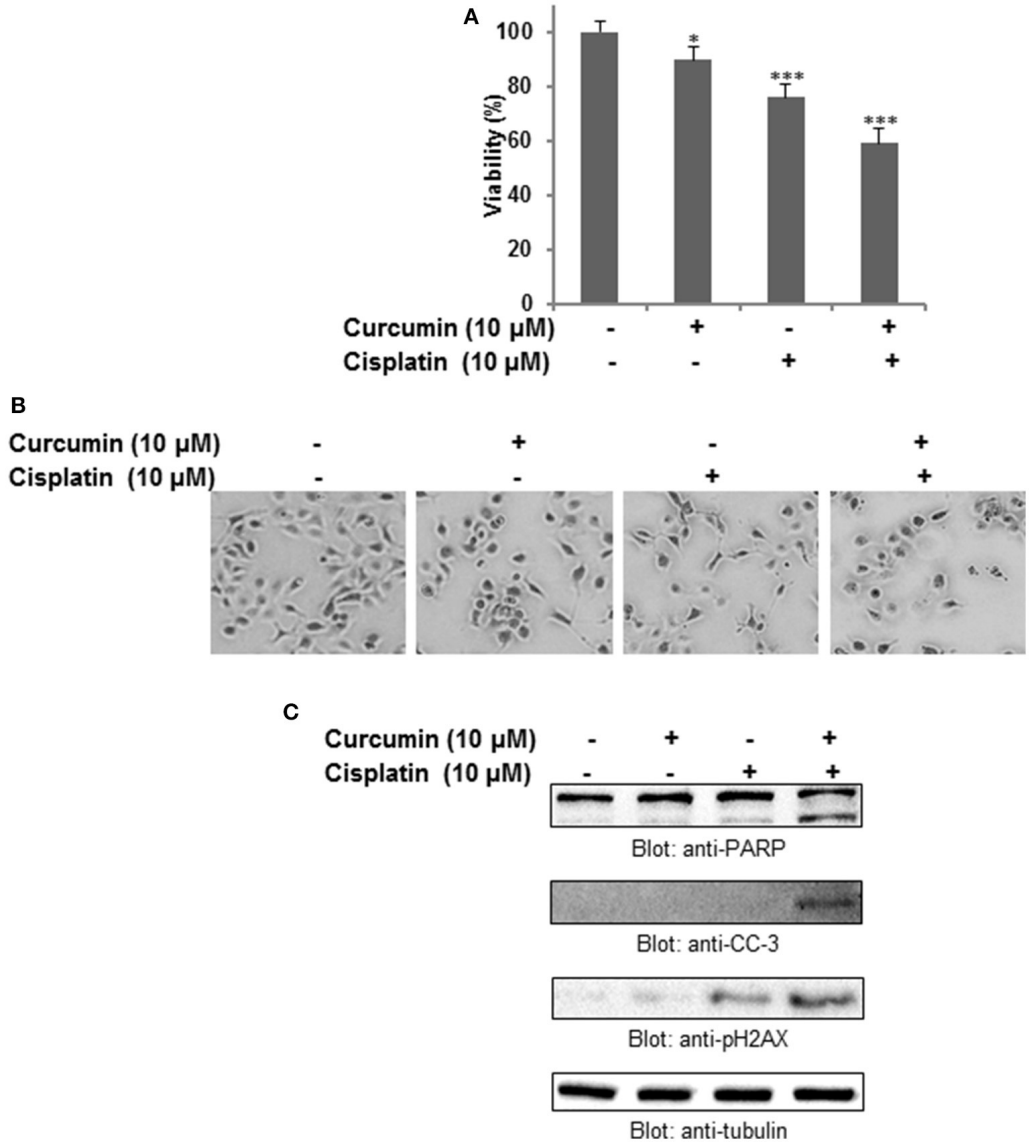
Curcumin augments the antitumor effect of cisplatin in HNSCC cells. **(A)** Combination treatment of curcumin and cisplatin potentiated inhibition of cell proliferation of HNSCC cells. FaDu cells were treated either with 10 μM curcumin and 10 μM cisplatin alone or with a combination of 10 μM curcumin and 10 μM cisplatin for 24 h. Cell proliferation assays were performed using CCK8 as described in Materials and methods. The graph displays the mean ± S.D. of three independent experiments with replicates of four wells for all the doses and vehicle control for each experiment. ^*^p < 0.05, ^***^p < 0.001. **(B)** Combination treatment of curcumin and cisplatin induced shrinkage (apoptosis) of HNSCC cells. **(C)** Combination treatment of curcumin and cisplatin potentiates activation of caspase, PARP and p-H2AX in HNSCC cell. FaDu cells were treated either with 10 μM curcumin or 10 μM cisplatin alone or with a combination of 10 μM curcumin and 10 μM cisplatin for 24 h. Cells were subsequently lysed, equal amounts of proteins were separated by SDS–PAGE and immunoblotted with antibodies against PARP, cleaved capsase-3, p-H2AX and tubulin as indicated.

To investigate whether the anticancer effect of curcumin can potentiate well-known chemotherapeutic drug such as cisplatin, we treated HNSCC cell lines with subtoxic doses of cisplatin and curcumin alone and in combination. It was observed that curcumin, in combination with cisplatin, showed remarkable action with respect to the cell viability, and apoptosis. As shown in Figure 6A, the combination of curcumin and cisplatin reduced cell proliferation significantly (*p* < 0.05, *p* < 0.001).This phenomenon was observed to be significantly higher as compared to curcumin or cisplatin alone. In the next series of experiments, we evaluated the effect of curcumin and cisplatin alone or in combination with these drugs on induction of apoptosis (cell shrinkage) in HNSCC cells (Figure 6B). It was observed that combination treatment of FaDu cells (curcumin and cisplatin) resulted in robust cleavage of PARP, activation of caspase 3 and phosphorylation H2AX (Figure 6C) suggesting that this combination potentiates a higher apoptotic response as compared to single drug treatment.

## Discussion

Head and neck squamous cell carcinoma (HNSCC) is one of the leading cancers worldwide. Tobacco use and alcohol consumption has been linked to cause for the development of HNSCC. In addition, involvement of HPV infection has been found to be associated with HNSCC (29). Recently, it has been reported that HPV positive patients showed a better prognosis (8). This notion of HNSCC implicate that due to better prognosis, HPV^+^ tumor cells possess intrinsic properties including an increased sensitivity to therapeutic agents, suppressed proliferation rate due to the presence of the virus. Uncontrolled cell proliferation, a crucial hallmark in carcinogenesis, is one of the main concerns in cancer management as a series of associated signaling molecules have been discovered and documented as putative targets for cancer treatment at different stages of drug development. Skp2 and associated signaling proteins are one of the major, studied target proteins of recent times for neoplastic therapy. Skp2, an F-box protein of SCF E3 ubiquitin ligase complex, known to have a critical role in growth and development as it regulates cell cycle, proliferation, differentiation, and survival which reflects Skp2 as a crucial target for anticancer drug development (18, 21, 22). Skp2 overexpression has been reported in various human malignancies including the head and neck and is known to exert its oncogenic action via degradation of its targets such as p27, p21, p57, and foxo1 via ubiquitinated degradation (14, 18).

In this study, we investigated the therapeutic potential of curcumin, a natural compound on HNSCC with and without containing HPV. We aimed to determine whether curcumin has differential targeting effects on Skp2 in HPV^+^ and HPV^−^ HNSCC cell lines. Our results showed that curcumin suppresses the cell viability in HNSCC cell lines, SCC25 and FaDu (HPV^−^) and SCC090 (HPV^+^) indicating that curcumin effect is independent of HPV status. It was observed that curcumin mediated inhibition of cell viability was due to apoptosis. A similar pattern of apoptosis was seen in both HPV^−^ and HPV^+^ cell lines.

Our findings revealed that curcumin downregulated the expression of Skp2 in three HNSCC cell lines SCC25 and FaDu and SCC090 in a dose dependent manner with concomitant elevated level of cyclin-dependent kinase inhibitors p27 and p21 expression. Our results support the hypothesis of inverse expression level between Skp2 and CDKIs. As a member of Fbox family proteins, Skp2 induced degradation of p27 and p21 via ubiquitination which was found evident from the findings of the current study. The cycloheximide mediated protein chase experiment revealed that curcumin stabilizes p27 expression which provides mechanistic evidence for the strong anti-proliferative potential of curcumin. siRNA mediated knockdown of Skp2 in FaDu and SCC090 cell lines depicts that curcumin strongly inhibits the growth of cancer cells via inactivation of Skp2 mediated degradation of CDKIs p27 and p21 most likely by apoptosis.

Apoptosis or programmed cell death is a complex and multistep process and play a vital role in maintaining the normal homeostatic function of cellular and physiological machinery. Apoptosis take place either through extrinsic (receptor mediated) pathway or the intrinsic pathway (mitochondrial-mediated) in mammalian cells (30). Most of the anticancer drugs induced apoptosis via mitochondrial or intrinsic apoptotic pathway (31). Curcumin has well-established apoptosis induction potential in a number of malignant cell types but not in normal cell types which suggest that curcumin could be a strong ideal candidate for anti-cancer drug development (27, 32–34). Our data reveals that curcumin causes dose-dependent inhibition of growth and proliferation of HNSCC in HPV^+^ and HPV^−^ cells via induction of the signaling proteins associated with apoptosis. Curcumin suppresses the expression of Bcl2 an antiapoptotic protein and enhanced the expression of Bax, a proapoptotic member of the protein. Elevated level of Bax and low level of Bcl2 has been shown to damage the mitochondrial membrane (35). Our data showed that curcumin treatment of SCC25, FaDu and SCC090 cell lines resulted in a loss in mitochondrial membrane potential as well as release of in cytochrome c release from mitochondrial to cytosol in all HNSCC cell lines. In cytosole, cytochrome forms a complex known as apoptosome via interaction of cytochrome C, apoptosome protease activating factor (APAF-1) and caspase-9. The apoptosome then leads to activation of caspase-9 and its downstream substrates caspase-3. Then activated caspase-3 resulted in cleavage and activation of PARP in execution of apoptotic cell death (36). Curcumin treatment resulted in activation of caspase-9, caspase-3, and cleavage of PARP ultimately resulting in DNA fragmentation and cell death. Curcumin-mediated overexpression of H2AX, a prominent marker of DNA strands break, reveals its apoptosis induction potential and thus suggest anti-proliferative and suppressive growth feature which is imperative for cancer treatment. The end point of apoptosis involves suppression of inhibitor of apoptosis proteins (IAPs). IAPs have been shown to prevent induction of apoptosis via inhibition of the activation and cleavage of the caspases proteins (37, 38). Therefore, downregulation of IAPs can leads to efficient apoptotic cell death. Our data showed that curcumin treatment of HNSCC cells downregulated IAPs member including XIAP, cIAP1, and cIAP2 in a dose dependent manner. Finally, we have also shown that curcumin potentiated the apoptotic effects of conventional chemotherapeutic agent cisplatin in HNSCC cells.

In summuary, findings of the current study reveal the mechanistic anti-tumorigenic action of curcumin in HNSCC independent of HPV status. Curcumin-mediated inhibition in the growth and proliferation of HNSCC cells is most likely through the inactivation of Skp2 mediated degradation of cyclin-dependent kinase inhibitor proteins via activation of mitochondrial apoptotic-caspase signaling pathways. Altogether, these data suggest a novel function for curcumin, acting as a suppressor of oncoprotein Skp2 in squamous cell carcinoma cells, and raise the possibility that this agent may have a future therapeutic role in squamous cell carcinoma and possibly other malignancies.

## Author contributions

SU and AK, experimental designing, data analysis, and manuscript writing. KS, KP, SK, SA, AS, and AR designing of experiments, data analysis, manuscript writing, and editing. FM and SD provided support in maintenance of cell culture and proofread of the manuscript.

### Conflict of interest statement

The authors declare that the research was conducted in the absence of any commercial or financial relationships that could be construed as a potential conflict of interest. The handling editor declared a shared affiliation, though no other collaboration, with one of the authors FM.

## Supplementary material

Supplementary Figure 1 Statistical analysis of curcumin mediated phosphorylation of H2AX in HNSCC cell lines. The graph displays the mean ± S.D. of three independent experiments for all the doses. ^**^ *p* < 0.01, ^***^ *p* < 0.001. **(A)** SCC25, **(B)** FaDu, and **(C)** SCC090 cells were treated with 10, 20, 40 μM curcumin for 24 h. Statistical analysis of curcumin modulated expression of Skp2, p27, p21 in HNSCC cell lines (mean ± S.D). **(D)** SCC25, **(E)** FaDu, and **(F)** SCC090 cells were treated with 10, 20, 40 μM curcumin for 24 h. ^*^*p* < 0.05, ^**^*p* < 0.01, ^***^*p* < 0.001.

Supplementary Figure 2 **(A)** Immunostaining of Skp2 of curcumin treated HNSCC cell lines. HNSCC cell lines, SCC25, FaDu, and SCC090 cells were treated with 20 μM curcumin for 24 h followed by fixation, immunostaining and imaging. **(B)** Curcumin treatment of HNSCC cells causes the stabilization of p27. FaDu cells were treated with and without 20 μM of curcumin for 24 h. Cells were then treated with 10 μM cycloheximide for 30, 60, 120, and 240 min. Cells were lysed and equal amounts of proteins were separated by SDS-PAGE, transferred to PVDF membrane, and immuno-blotted with antibodies against p27 and GAPDH as indicated.

Supplementary Figure 3 Curcumin down-regulates expression of inhibitors of apoptotic proteins (IAPs) in HNSCC cell lines. **(A)** SCC25, **(B)** FaDu, and **(C)** SCC090 cells were treated with 10, 20, and 40 μM curcumin for 24 h. Following treatment, cells were harvested and proteins were isolated and separated on SDS-PAGE and immunoblotted with antibodies against XIAP, cIAP1, cIAP2, and GAPDH as indicated.
