# Corrigendum: Curcumin-mediated degradation of S-phase kinase protein 2 induces cytotoxic effects in human papillomavirus-positive and negative squamous carcinoma cells

**DOI:** 10.3389/fonc.2023.1292898

**Published:** 2023-09-28

**Authors:** Abdul Q. Khan, Kodappully S. Siveen, Kirti S. Prabhu, Shilpa Kuttikrishnan, Sabah Akhtar, Abdullah Shaar, Afsheen Raza, Fatima Mraiche, Said Dermime, Shahab Uddin

**Affiliations:** ^1^ Translational Research Institute, Academic Health System, Hamad Medical Corporation, Doha, Qatar; ^2^ National Center for Cancer Care and Research, Hamad Medical Corporation, Doha, Qatar; ^3^ School of Pharmacy, Qatar University, Doha, Qatar

**Keywords:** cancer, head and neck squamous cell carcinoma, HPV, Skp_2_, apoptosis

In the published article, there was an error in the Funding statement. The grant number included was incorrect. The original text incorrectly states that “This work was supported by Medical Research Centre (Grant No. RP # 16354/16), Hamad Medical Corporation, Doha, Qatar. The publication of this article was funded by the Qatar National Library”. The correct Funding statement appears below.

**FUNDING**

This work was supported by Medical Research Centre (Grant No. MRC-01-18-186), Hamad Medical Corporation, Doha, Qatar. The publication of this article was funded by the Qatar National Library.

The authors apologize for this error and state that this does not change the scientific conclusions of the article in any way.

