# Supplementary figures and images for: Curcumin-Mediated Degradation of S-Phase Kinase Protein 2 Induces Cytotoxic Effects in Human Papillomavirus-Positive and Negative Squamous Carcinoma Cells
Source: Front Oncol. 2018 Oct 2;8:399. doi: 10.3389/fonc.2018.00399 (PMC6176276; doi:10.3389/fonc.2018.00399)

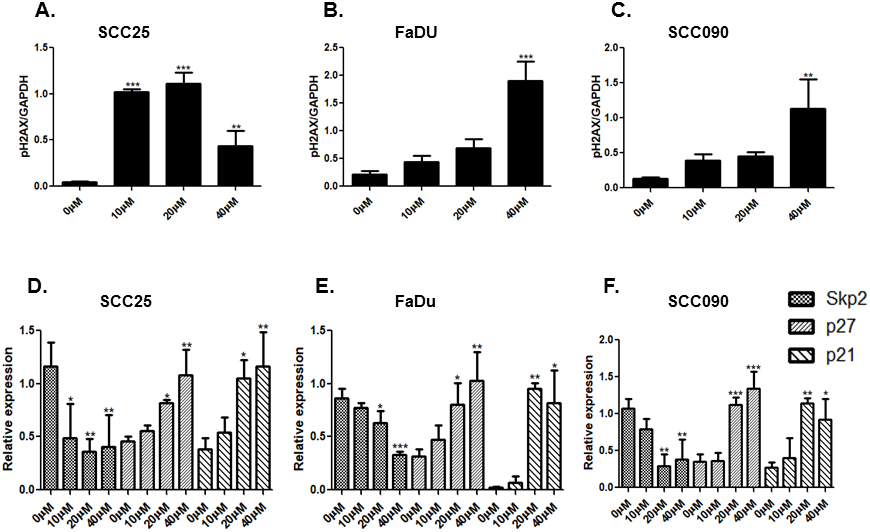

Supplement: Supplementary Figure 1 — Statistical analysis of curcumin mediated phosphorylation of H2AX in HNSCC cell lines. The graph displays the mean ± S.D. of three independent experiments for all the doses. ** p < 0.01, *** p < 0.001. (A) SCC25, (B) FaDu, and (C) SCC090 cells were treated with 10, 20, 40 μM curcumin for 24 h. Statistical analysis of curcumin modulated expression of Skp2, p27, p21 in HNSCC cell lines (mean ± S.D). (D) SCC25, (E) FaDu, and (F) SCC090 cells were treated with 10, 20, 40 μM curcumin for 24 h. *p < 0.05, **p < 0.01, ***p < 0.001. [file Image_1.tif]

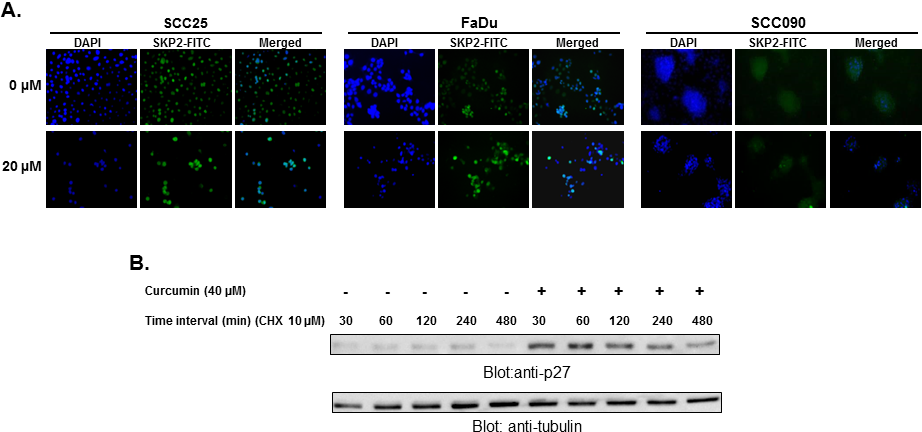

Supplement: Supplementary Figure 2 — (A) Immunostaining of Skp2 of curcumin treated HNSCC cell lines. HNSCC cell lines, SCC25, FaDu, and SCC090 cells were treated with 20 μM curcumin for 24 h followed by fixation, immunostaining and imaging. (B) Curcumin treatment of HNSCC cells causes the stabilization of p27. FaDu cells were treated with and without 20 μM of curcumin for 24 h. Cells were then treated with 10 μM cycloheximide for 30, 60, 120, and 240 min. Cells were lysed and equal amounts of proteins were separated by SDS-PAGE, transferred to PVDF membrane, and immuno-blotted with antibodies against p27 and GAPDH as indicated. [file Image_2.tif]

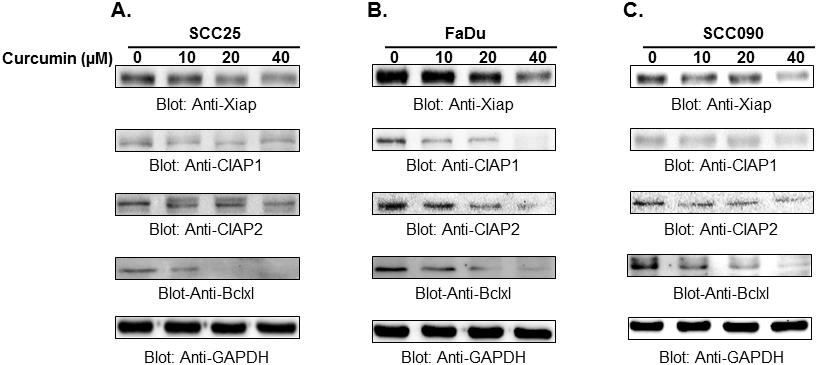

Supplement: Supplementary Figure 3 — Curcumin down-regulates expression of inhibitors of apoptotic proteins (IAPs) in HNSCC cell lines. (A) SCC25, (B) FaDu, and (C) SCC090 cells were treated with 10, 20, and 40 μM curcumin for 24 h. Following treatment, cells were harvested and proteins were isolated and separated on SDS-PAGE and immunoblotted with antibodies against XIAP, cIAP1, cIAP2, and GAPDH as indicated. [file Image_3.tif]
